# PE_PGRS33 Contributes to *Mycobacterium tuberculosis* Entry in Macrophages through Interaction with TLR2

**DOI:** 10.1371/journal.pone.0150800

**Published:** 2016-03-15

**Authors:** Ivana Palucci, Serena Camassa, Alessandro Cascioferro, Michela Sali, Saber Anoosheh, Antonella Zumbo, Mariachiara Minerva, Raffaella Iantomasi, Flavio De Maio, Gabriele Di Sante, Francesco Ria, Maurizio Sanguinetti, Giorgio Palù, Michael J. Brennan, Riccardo Manganelli, Giovanni Delogu

**Affiliations:** 1 Institute of Microbiology, Università Cattolica del Sacro Cuore, L.go A. Gemelli, 8–00168, Rome, Italy; 2 Institute of General Pathology, Università Cattolica del Sacro Cuore, L.go A. Gemelli, 8–00168, Rome, Italy; 3 Department of Molecular Medicine, University of Padua, Via A. Gabelli, 63–35121, Padua, Italy; 4 Aeras, Rockville (MD), United States of America; Bose Institute, INDIA

## Abstract

PE_PGRS represent a large family of proteins typical of pathogenic mycobacteria whose members are characterized by an N-terminal PE domain followed by a large Gly-Ala repeat-rich C-terminal domain. Despite the abundance of PE_PGRS-coding genes in the *Mycobacterium tuberculosis* (*Mtb*) genome their role and function in the biology and pathogenesis still remains elusive. In this study, we generated and characterized an *Mtb* H37Rv mutant (*Mtb*Δ33) in which the structural gene of PE_PGRS33, a prototypical member of the protein family, was inactivated. We showed that this mutant entered macrophages with an efficiency up to ten times lower than parental or complemented strains, while its efficiency in infecting pneumocytes remained unaffected. Interestingly, the lack of PE_PGRS33 did not affect the intracellular growth of this mutant in macrophages. Using a series of functional deletion mutants of the PE_PGRS33 gene to complement the *Mtb*Δ33 strain, we demonstrated that the PGRS domain is required to mediate cell entry into macrophages, with the key domain encompassing position 140–260 amino acids of PE_PGRS33. PE_PGRS33-mediated entry into macrophages was abolished in TLR2-deficient mice, as well as following treatment with wortmannin or an antibody against the complement receptor 3 (CR3), indicating that PE_PGRS33-mediated entry of *Mtb* in macrophages occurs through interaction with TLR2.

## Introduction

Tuberculosis (TB) is still one of the most relevant public health issues worldwide and primarily in developing countries where it poses a tremendous health, economic and social burden [1]. Decisive prophylactic and therapeutic tools are still missing, in part because many aspects of TB pathogenesis and of *Mycobacterium tuberculosis* (*Mtb*) biology remain elusive [2,3]. In the last two decades, a new understanding of this ancient disease has been gained following the deciphering of the *Mtb* genome [4], which uncovered unexpected and surprising features of the tubercle bacilli [5,6]. The disclosure that almost 10% of the *Mtb* genome coding capacity is devoted to the mycobacterial-specific PE and PPE protein families raised much interest on their role in *Mtb* biology [7–10].

All PE proteins are characterized by a highly conserved N-terminal domain of ≅ 100 amino acids with the presence of a proline-glutamic acid (PE) motif at position 8–9 [4]. Out of the 99 PE genes found in *Mtb* H37Rv [4], 63 were annotated as PE_PGRS though some of these were pseudogenes or lacked some of the typical PE_PGRS features, so that only 51 PE_PGRS potentially functional proteins are expressed [8]. PE_PGRSs proteins share the same molecular architecture, characterized by the presence, beyond a PE domain, of *i*) a typical PGRS domain varying in sequence and size containing a variable number of GGA-GGN repeats; *ii*) a highly conserved putative transmembrane domain linking the PE and PGRS domains with a GRPLI motif around position 115; *iii*) a unique C-terminal domain which is usually less than 30 amino acids long, but that in some cases (such as in PE_PGRS30) can be as large as 300 amino acids [7,11,12]. PE_PGRS-encoding genes are found only in few mycobacterial species (*M*. *marinum*, *M*. *ulcerans* and members of the *Mtb* complex), are scattered throughout the genome, and are differently regulated. The transcriptional regulation of some of them appears to be finely tuned depending on the environmental signals encountered during the complex steps of the infectious process, while others (as that encoding PE_PGRS33) are constitutively expressed [7,8,13,14]. The paucity of experimental data on PE_PGRSs has so far hampered a sufficient understanding of their role in TB pathogenesis.

PE_PGRS33 (Rv1818c), which can be considered a model for the family, is a 498 amino acids protein whose PE domain mediates its translocation on the mycobacterial outer membrane [15], and was used to deliver antigens on the mycobacterial surface [16–18]. Its surface localization makes it available for interaction with host components, as suggested by studies carried out with the avirulent vaccine strain *Mycobacterium bovis* BCG [19]. Interestingly, PE_PGRS33 was previously shown to trigger macrophage cell death by inducing secretion of pro-inflammatory cytokines [20,21] and activation of pro-apoptotic or pro-necrotic signals involving mitochondria [22–24]. Basu *et al*. [23] showed that PE_PGRS33 was capable of inducing TLR2-dependent apoptosis in macrophages. All the studies aimed at investigating the pro-inflammatory role of PE_PGRS33 were carried out by ectopically expressing PE_PGRS33 in the avirulent species *M*. *smegmatis* [20,21], by directly using the purified recombinant protein obtained in *Escherichia coli* [22,23] or by expressing the mycobacterial protein directly in host cells using an eukaryotic expression plasmids [24].

In this study, we generated an *Mtb* Rv1818c null mutant, which was complemented with a series of Rv1818c genes manipulated to functionally dissect PE_PGRS33 structure. The resulting strains were used to investigate the role of PE_PGRS33 in *Mtb* cell entry gaining insights in the involvement of this protein in TB pathogenesis.

## Material and Methods

### Bacterial Strains

*Mtb* was grown at 37°C in Middlebrook 7H9 or 7H10 (Difco Becton-Dickinson), supplemented with 0.2% glycerol (Sigma-Aldrich), ADC 10% (Becton-Dickinson), and 0.05% v/v Tween 80 (Sigma-Aldrich). The PE_PGRS33 mutant was generated in *Mtb* H37Rv by allelic exchange using the recombineering system [25]. Briefly, we constructed a pJSC-derivative vector, in which a region upstream Rv1818c (-563 to -1 bp) and a region internal to its coding sequence (561 to 1004 bp) were cloned at the flanks of an hygromycin cassette. The resulting recombination substrate was digested, purified and introduced by electroporation in *Mtb* H37Rv competent cells containing pJV53, carrying the recombinases and conferring kanamycin resistance. Transformants were first selected on 7H11/OADC-Tween 80 plates containing hygromycin (50 μg ml^-1^) at 37°C for 3–4 weeks and selected colonies analyzed by PCR to demonstrate Rv1818c deletion (S1 Fig). One mutant with the correct deletion was sub-cultured in drug-free media for about 10 generations to allow the loss of pJV53 and then plated on solid medium containing hygromycin at 37°C. Single colonies were picked and analyzed for loss of the kanamycin resistance to isolates a mutant without pJV53. The Rv1818c null mutant was then complemented using the integrative pMV306-derivative pAL79 [17] containing a copy of Rv1818c gene fused with the sequence encoding the HA epitope under the control of its own putative promoter (S2 Fig) [16].

### Generation of PE_PGRS33 Functional Deletion Mutants for the PGRS Domain

The *Mtb* H37Rv PE_PGRS33 hydrophobicity profile was obtained by *in silico* analysis using the Kyte-Doolittle scale (CLC Main Workbench 6.9). Based on the hydrophobic/hydrophilic characteristics at the C-terminal PGRS domain, four Rv1818c fragments encoding the first 472, 401, 341 and 260 amino acids, lacking variable size of the PGRS domain, were amplified from the *Mtb* H37Rv genome (using primers indicated in S1 Table) and cloned under the control of the Rv1818c native promoter into the integrative plasmid pMV306 upstream and in frame with the HA epitope sequence.

### Cell Line Culture

The J774 cell line (murine macrophage) were grown in RPMI 1640 medium containing 10% fetal calf serum (FCS) supplemented with glutamine (2 mM), streptomycin (100 μg/ml), penicillin (100 U/ml), and sodium pyruvate (1 mM). Cells were kept in a humidified atmosphere containing 5% CO_2_ at 37°C. The cells were diluted to 1.2 x10^6^ per ml in 48 and 24-well plates with 2% FCS. Human THP-1 monocytic cells were grown in RPMI 1640 supplemented with glutamine (2 mM) and 10% FCS. Cells were treated with 20 nM PMA (Sigma-Aldrich, St. Louis, MO) for 24 h to induce their differentiation into macrophage-like cells, then washed three times with PBS and maintained in 2% FCS.

The human lung adenocarcinoma epithelial cell line, A549 (ATCC, Rockville, MD), were used as model of human type II alveolar epithelial cells. Cells were grown in complete medium consisting of RPMI 1640 supplemented with 10% FCS, 2 mM L-glutamine and 5 μg/ml of gentamicin, and split when a confluent cell monolayer was attained [26].

### Primary Murine Peritoneal Macrophages (*pMM0*)

C57BL/6 mice were purchased from Harlan (Italy) and maintained in pathogen-free micro-isolator cages. The animals were housed in a temperature-controlled environment with 12 h light/dark cycles, and received food and water ad libitum. All animal experiments were authorized by the Ethical Committee of the Università Cattolica del Sacro Cuore (n°T21/2011) and performed in compliance with the legislative decree of the Italian Government 27 January 1992, n. 116 and the Health Minister memorandum 14 May 2001, n. 6. All manipulations were performed under isoflurane anesthesia, and all efforts were made to minimize suffering. Nine-fourteen weeks-old female mice were euthanized by inhalation of isoflurane (Baxter, Warsaw, Poland). Inflammatory peritoneal cells, elicited with 1 ml of aged 3% thioglycollate (Difco, Detroit, MI), injected intraperitoneal 4–5 days earlier, were washed out with phosphate buffered saline (PBS) and collected into centrifuge tubes kept on ice. After washing once, peritoneal cells were re-suspended in the RPMI/FBS medium and plated at 1.2×10^6^ cells/ml in 48-well tissue culture plates. Following overnight incubation, non-adherent cells were removed by washing, whereas adherent macrophages were used for experiments. Similar procedures were used to isolate *pMM0* from TLR2^-/-^ mice.

### Mycobacterial Infection and Bacterial Counts *In Vitro*

Cells were infected with mycobacteria at different multiplicity of infection (MOI) and incubated for 4 hours post-infection at 37°C in a 5% CO_2_ atmosphere. Cells were then washed three times with PBS to remove extracellular bacteria and then incubated with complete RPMI 2% of FCS without antibiotics. To assess the ability of the *Mtb* strains to survive intracellularly, macrophages were infected at different MOIs (1:10, 1:1 or 10:1) and infection in A549 cells was carried at MOI of 5:1. Cells were washed with PBS three times to remove extracellular bacteria and then incubated in fresh medium. At different time points, cells were lysed in 0.1% of Triton X-100 and intracellular bacteria determined by CFU counting by serially diluting lysates in PBS containing Tween80 (0.05%) and plating on 7H11/OADC agar plates [13]. Colony counting was then performed in triplicate. In some cases, after infection the supernatant, containing not internalized bacteria was collected and plated on 7H11/OADC agar plates.

*pMM0* were treated before infection with wortmannin (Calbiochem, San Diego, CA) at the concentration of 100 nM and 300 nM and with mAb anti-CR3 at final concentration of 10 μg/ml (clone M1/70 BD, Biosciences) for 30 minutes at 37°C and then infected with *Mtb* strains at MOI 1:10 and intracellular bacteria counted as above. Statistical analysis to assess differences between the *Mtb*Δ33 mutants strains and parental and complemented strains was performed using the Student’s *t*-test and ANOVA with GraphPad Prism software version 5.0 (GraphPad software, CA, USA).

## Results

### Generation of an Rv1818c Mutant and Its Complementation

To investigate the role of PE_PGRS33 in *Mtb* pathogenesis, inactivation of the Rv1818c gene by recombineering [25] was carried out on the *Mtb* H37Rv strain as shown in S1 Fig, to generate the *Mtb*Δ33 mutant strain. The lack of an antibody specific for PE_PGRS33 prevented the demonstration of the gene inactivation by immunoblot [27]. The mutant strain was complemented by transformation with an integrative plasmid containing Rv1818c fused with the sequence encoding the HA epitope, expressed from its own promoter [16,17] (S2 Fig). Expression of the PE_PGRS33^HA^ in the *Mtb*Δ33 complemented strain (*Mtb*Δ33::PE_PGRS33^HA^) was demonstrated by immunoblotting using an anti-HA antibody (S2 Fig). Conversely, from what previously observed with the BCG mc^2^1525 mutant, where inactivation of the Rv1818c was obtained by transposon mutagenesis [19], no significant differences in the growth features of the *Mtb* H37Rv, *Mtb*Δ33 and *Mtb*Δ33::PE_PGRS33^HA^ in solid media and liquid culture were observed (data not shown). In any case, the lack of a phenotype in axenic culture for the *Mtb*Δ33 mutant provided a reliable model to investigate the role of PE_PGRS33 in *Mtb* cell entry.

### PE_PGRS33 Is Required for *Mtb* Cell Entry into Macrophages

**Fig 1:**
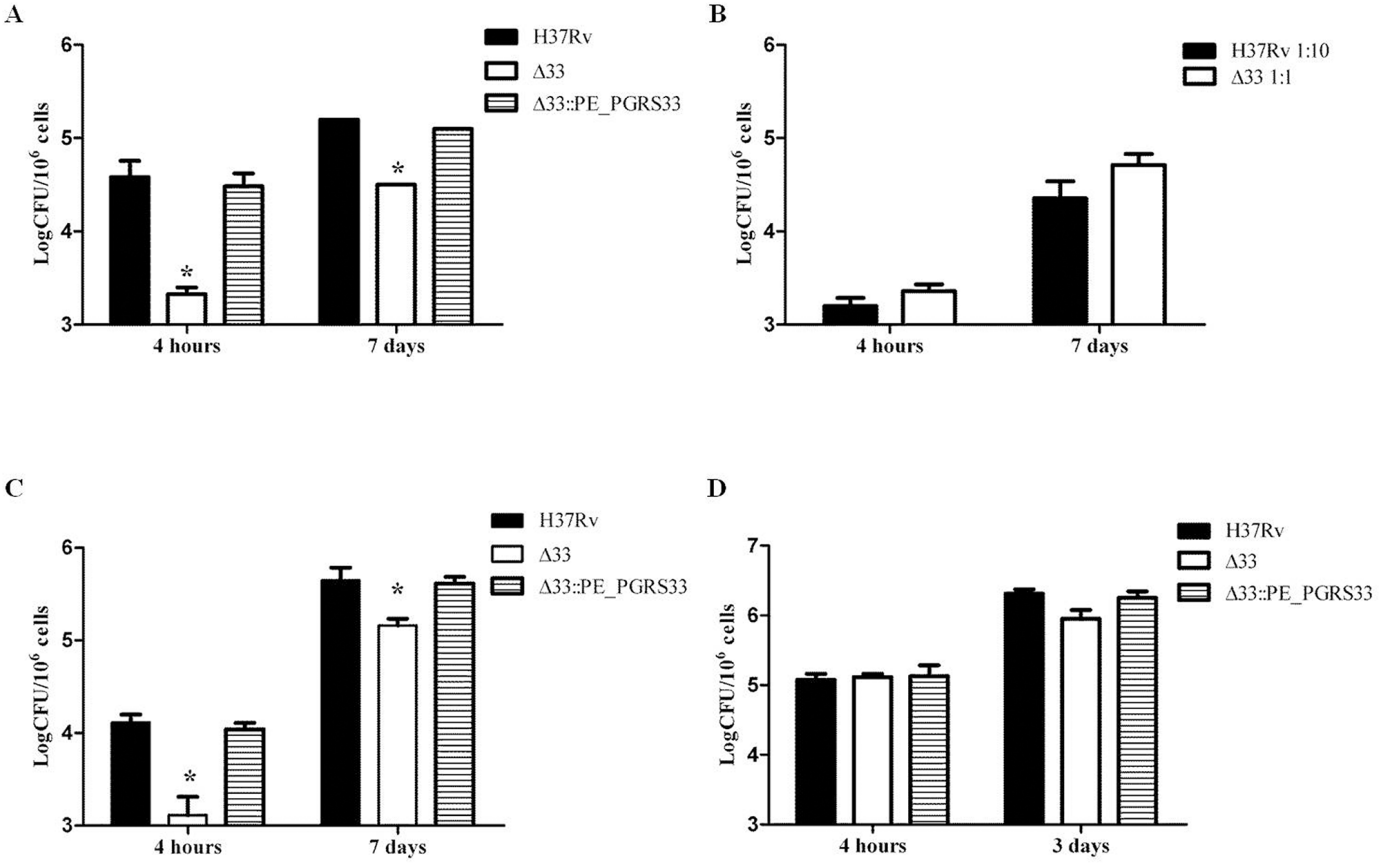
*Mtb*Δ33 is impaired in its ability to enter macrophages. Murine J774 macrophages (A and B), human monocyte-derived macrophages THP-1 (C) and human type II pneumocytes A549 (D) were infected with *Mtb* H37Rv, *Mtb*Δ33 and *Mtb*Δ33::PE_PGRS33 strains at a MOI 1:10 (A-C) or 5:1 (D) and following incubation cells were washed and at the different time points intracellular bacteria were determined by CFU counting. In the experiment shown in panel B, the *Mtb* H37Rv was infected at MOI 1:10, while the *Mtb*Δ33 at MOI 1:1. Results of one representative experiments from at least three assays are shown (*p<0.,01). CFUs were expressed as mean ± SD and were analysed by two-way ANOVA followed by Bonferroni posttest.

Seminal studies showed that PE_PGRS33 is exposed on the surface and that lack of PE_PGRS33 in a BCG transposon insertion mutant prevents proper bacilli entry into host cells [19]. To assess whether this was also true for virulent *Mtb*, J774 murine macrophages were infected with H37Rv, the *Mtb*Δ33 mutant and the *Mtb*Δ33::PE_PGRS33^HA^ complemented strain. The mutant strain was strongly impaired in its ability to enter macrophages by a reduction of 1.3 Log CFU of intracellular bacteria after 4h of infection compared to wild type strain, (p<0.01), although the intracellular replication rate of the mutant that entered macrophages was similar to that of the parental or complemented strains (Fig 1A). Interestingly, when the infection was repeated increasing the multiplicity of infection (MOI) of the *Mtb*Δ33 mutant, keeping the number of the parental *Mtb* strain constant, at 4h post-infection a similar number of intracellular bacteria for the two strains was found and their number increased after 7 days of infection (Fig 1B).

The role of PE_PGRS33 in *Mtb* entry was also confirmed in human monocyte-derived macrophages (THP-1), with *Mtb*Δ33 impaired in its ability to enter these cells (Fig 1C; - 0.99 Log CFU in the *Mtb*Δ33 compared to the *Mtb* H37Rv parental strain; p<0.01), yet invading organisms were still capable of intracellular replication as shown by intracellular CFUs measured at day 7 (Fig 1A–1C). Taken together these results indicate that PE_PGRS33 significantly contributes to the ability of *Mtb* to enter macrophages, but is dispensable for *Mtb* intracellular replication.

Interestingly, when the same experiments were performed in type II human pneumocytes (A549) following usual experimental settings [26], no differences in the ability to infect these cells were observed among the three strains (Fig 1D), suggesting that PE_PGRS33 is required for *Mtb* entry in macrophages but not in epithelial cells.

**Fig 2:**
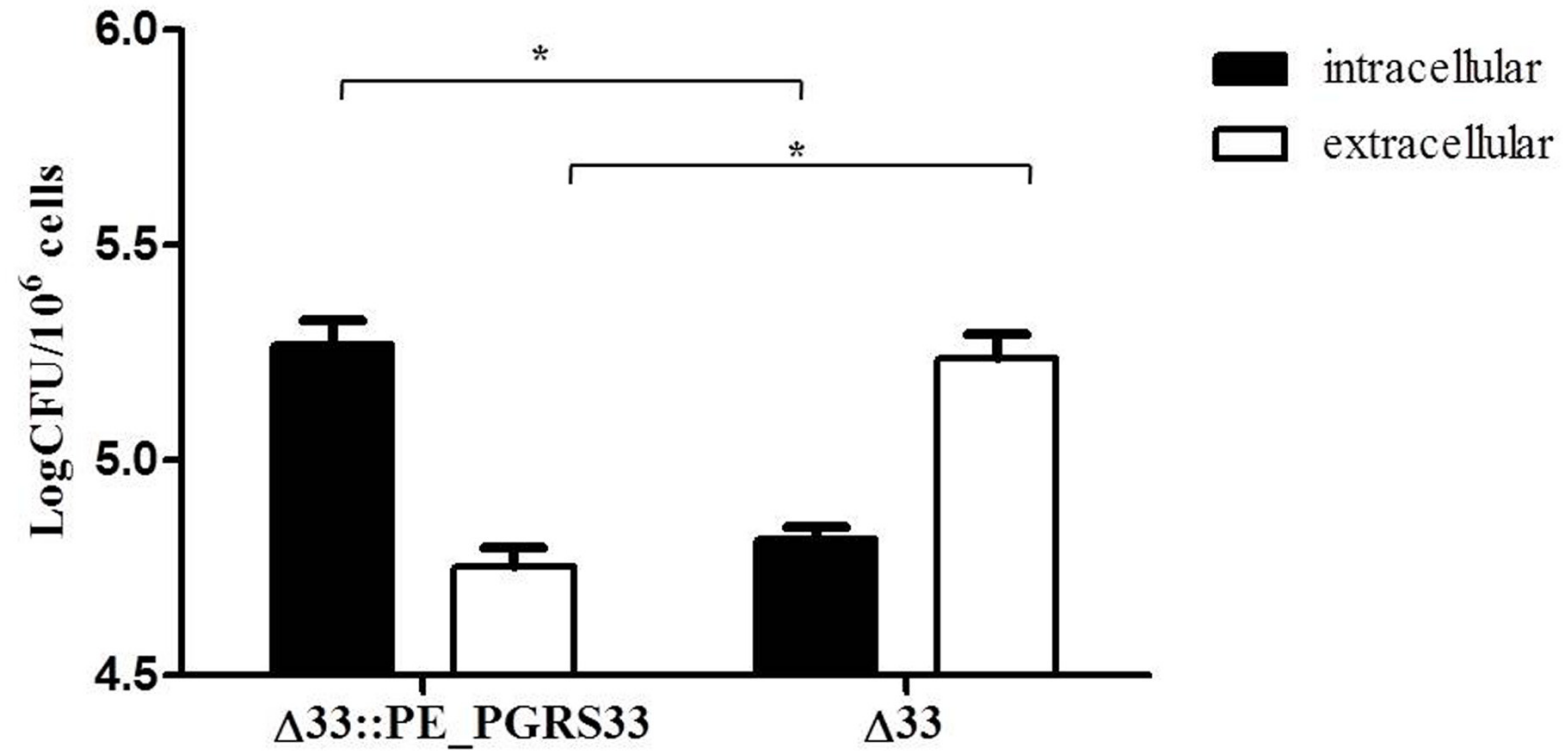
Deletion of PE_PGRS33 gene is associated with a defect in bacterial entry in *pMM0*. *pMM0* were infected with *Mtb* H37Rv, *Mtb*Δ33 and *Mtb*Δ33::PE_PGRS33 strains at MOI 1:1 to measure the number of intracellular vs extracellular bacteria at 4h post infection. Results of one representative experiments from at least three assays are shown (*p<0.05). CFUs were expressed as mean ± SD and were analysed by two-way ANOVA followed by Bonferroni posttest.

To further investigate the role of PE_PGRS33 in *Mtb* cell entry, murine peritoneal macrophages (*pMM0*) from C57Bl/6 mice were infected with *Mtb* H37Rv, *Mtb*Δ33 and *Mtb*Δ33::PE_PGRS33^HA^ and the number of intracellular and extracellular bacilli was assessed by CFU counting. As shown in Fig 2, while most of the *Mtb*Δ33::PE_PGRS33^HA^ bacilli were detected intracellularly, the *Mtb*Δ33 mutant was primarily detected in the supernatant and an inverse correlation among the two *Mtb* strains was observed, clearly indicating that viable *Mtb*Δ33 mutant organisms are indeed impaired in their ability to enter these macrophages.

### PE_PGRS33-Mediated *Mtb* Cell Entrance in Macrophages Occurs through Interaction with TLR2

**Fig 3:**
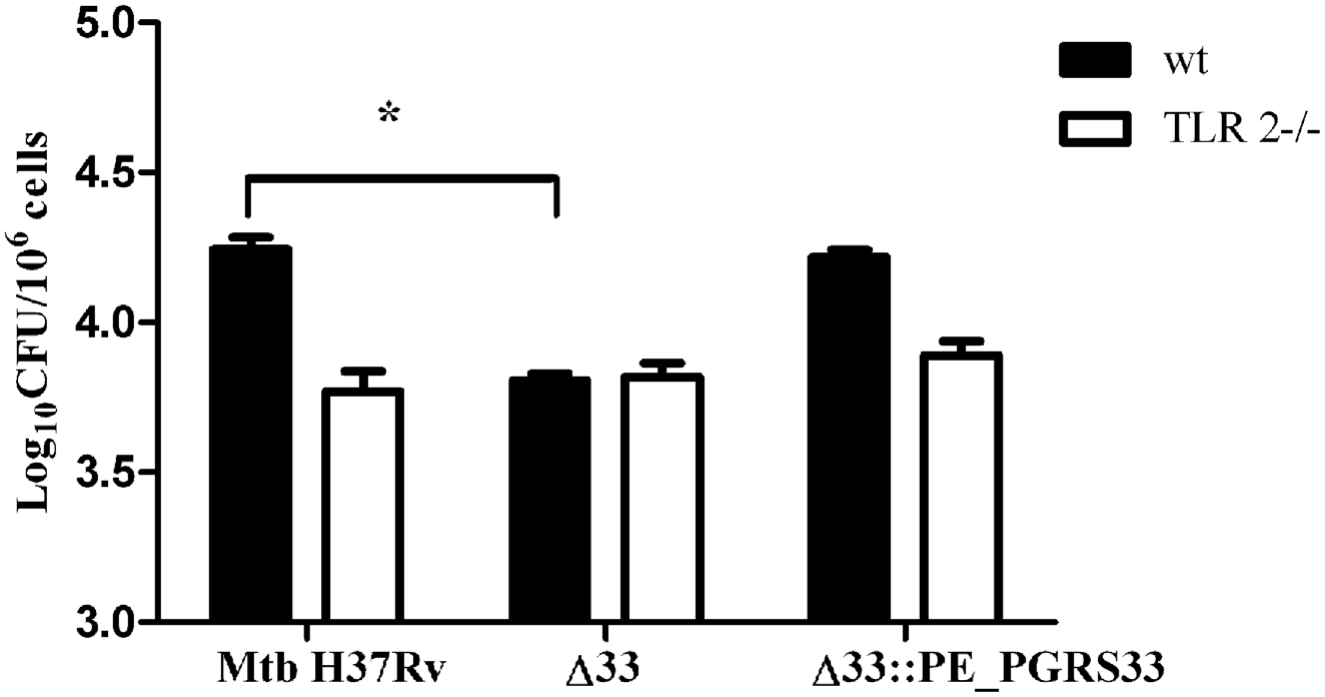
PE_PGRS33-mediated *Mtb* cell entry in macrophages occurs through interaction with TLR2. *pMM0* isolated from C57Bl/6 and syngeneic TLR2^-/-^ mice were infected with *Mtb* H37Rv, *Mtb*Δ33 and *Mtb*Δ33::PE_PGRS33^HA^ and intracellular CFUs were determined at 4h post-infection. Results of one representative experiments of at least three assays are shown (**p<0,.01). CFUs were expressed as mean ± SD and were analysed by two-way ANOVA followed by Bonferroni posttest.

A number of mycobacterial components are known to interact with TLR2, such as lipoarabinomannan (LAM), lipomannan (LM), phosphatidil-myo-inositol mannoside (PIM), the 19-kDa lipoprotein [28]. The immunomodulatory properties of the mycobacterial surface protein PE_PGRS33 were found to be dependent upon the ability of the protein to bind and trigger TLR2 [22,23]. Interestingly, interactions of microbial ligands with TLR2 were shown to mediate entry of certain bacteria into macrophages [29]. To establish whether PE_PGRS33-mediated entrance of *Mtb* in macrophages was dependent on TLR2, *pMM0* isolated from wild type and TLR2-deficient mice were infected with H37Rv, *Mtb*Δ33 and the complemented strain. As shown in Fig 3, no differences were observed between *Mtb*Δ33 and the parental or complemented strains in their ability to enter in TLR2^-/-^ *pMM0*. These results demonstrate that PE_PGRS33 mediates entrance of *Mtb* in macrophages in a TLR2-dependent mechanism.

### PE_PGRS33 Activates the TLR2-Dependent Pro-Adhesive Pathway in Macrophages

**Fig 4:**
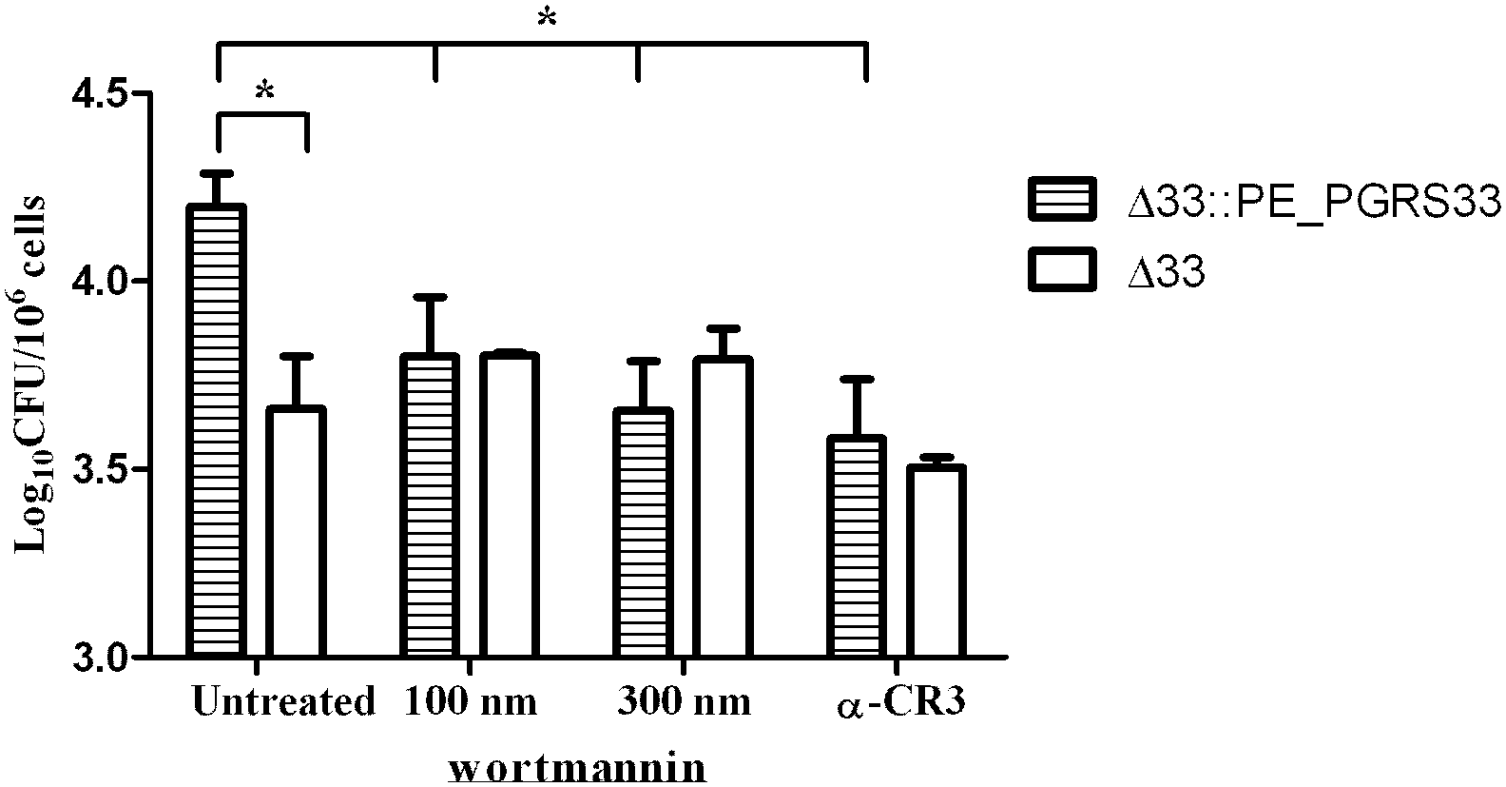
PE_PGRS33 activates the TLR2-dependent pro-adhesive pathway in macrophages. *pMM0* isolated from C57Bl/6 mice were incubated with wortmannin or with an anti-CR3 antibody (M1/70), or left untreated, and then infected (MOI 1:10) with *Mtb*Δ33 and the complemented strain *Mtb*Δ33::PE_PGRS33^HA^ and intracellular CFUs were determined at 4h post-infection. Results of one representative experiments of at least three assays are shown (*p<0,.001). CFUs were expressed as mean ± SD and were analysed by two-way ANOVA followed by Bonferroni posttest.

Interaction of bacterial surface components with TLR2 has been shown to trigger the pro-adhesive pathway by enhancing the avidity of the CR3 receptor through the activity of phosphatidylinositol 3-kinase (PI3K) [30,31]. To investigate whether this was also the case in the PE_PGRS33-mediated entrance of *Mtb*, *pMM0* were previously treated with wortmannin, an inhibitor of PI3K [31]. As shown in Fig 4, *pMM0* treated with wortmannin were impaired in their ability to phagocytize *Mtb*Δ33::PE_PGRS33^HA^, but not *Mtb*Δ33, compared to untreated *pMM0* indicating that inhibition of PI3K abolishes the PE_PGRS33-mediated entrance in macrophages. Equally, incubation of *pMM0* with an antibody directed against CR3 inhibited entrance of *Mtb*Δ33::PE_PGRS33^HA^, but not of *Mtb*Δ33 into macrophages (Fig 4) providing further evidences that the activation of TLR2 by PE_PGRS33 triggers the pro-adhesive pathway which enhances CR3 avidity for mycobacteria. The PE_PGRS33-mediated entrance of *Mtb* in macrophages was observed in opsonized and non-opsonized bacilli (data not shown) suggesting that this phenomena is not restricted for non-opsonic entrance [32]. Further characterization of the *Mtb*Δ33 mutant, including *in vivo* studies, will provide insights on the role of PE_PGRS33 in the early and late steps of the TB infectious process.

### The PGRS Domain Encompassing Position 140–260 of the Protein Is Responsible for PE_PGRS33-Mediated *Mtb* Entry into Macrophages

**Fig 5:**
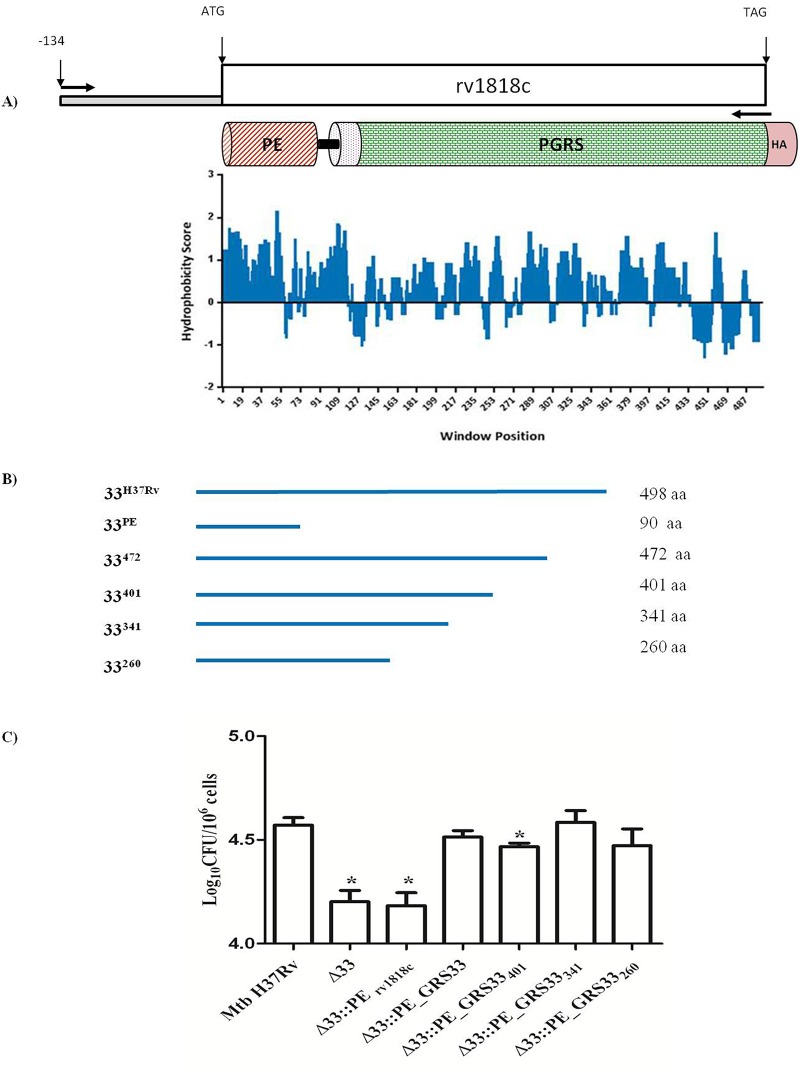
PE_PGRS33 encoding the first 260 amino acids is capable to mediate the entrance phenotype into macrophages. Schematic of PE_PGRS33 fragments (A-C). Truncated forms of Rv1818c were cloned downstream the native promoter PE_PGRS33 and in frame with the HA epitope sequence into the integrative plasmid pMV306. A) Rv1818c gene and its native putative promoter located 134 nucleotides upstream the start codon. Schematic overview showing PE_PGRS33 domains: PE domain (red stripped), transmembrane region (magenta dotted), PGRS domain (green netting), HA epitope (rose). The hydrophobicity profile of PE_PGRS33 was obtained using CLC Main Workbench 6.9 (Kyte-Doolittle scale, window size 9). B) Stylized representation of full-length PE_PGRS33, PE domain and the 472, 401, 341 and 260 amino acid in length fragments (blue lines). Cloning and protein information are reported beside. C) *pMM0* isolated from C57BL/6 mice were infected at a MOI of 1:10 with *Mtb* H37Rv, *Mtb*Δ33, *Mtb*Δ33::PE_PGRS33HA and complemented strains expressing the 4 PE_PGRS33 functional deletion mutants. Intracellular CFUs were enumerated at 4h post-infection. Results of one representative experiment of at least three assays are shown. * p<0.05 for *Mtb* H37Rv vs *Mtb*Δ33::PE_PGRS33 401 and *** p<0.001 compared with for *Mtb* H37Rv vs *Mtb*Δ33 and *Mtb*Δ33::PE Rv1818c. CFUs were expressed as mean ± SD and were analysed by one-way ANOVA followed by Dunnett’s multiple comparison test.

In an attempt to identify the functional region(s) of PE_PGRS33 implicated in the entry of *Mtb* into macrophages, we analyzed the protein hydrophobicity pattern (Fig 5A), which revealed the presence of numerous hydrophobic regions intercalated by shorter hydrophilic segments, primarily located in the PGRS domain and likely available on *Mtb* surface for interaction with TLR2 [16,17,23]. Based on these observations, four PE_PGRS33 gene fragments corresponding to the sequences encoding the first 472, 401, 341 and 260 amino acid residues of PE_PGRS33 (PE_PGRS33_472_, PE_PGRS33_401_, PE_PGRS33_341_, PE_PGRS33_260_) were cloned in an integrative plasmid under the transcriptional control of the native promoter. These sequences were inserted in frame with the HA epitope sequence to demonstrate protein expression of the functional deletion mutants by immunoblot (data not shown). These four plasmids and the plasmid encoding the functional mutant lacking the entire PGRS domain (PE_1818c_) [17] were used to complement the *Mtb*Δ33 strain (Fig 5B). *pMM0* cells were infected at a MOI of 1:10 with *Mtb* H37Rv, *Mtb*Δ33, *Mtb*Δ33::PE_PGRS33 and the panel of complemented strains and bacterial entry was measured by counting intracellular mycobacteria at 4 hours post-infection. As expected [17,23,33], complementation of the *Mtb*Δ33 mutant with the plasmid expressing the PE_1818c_ domain only did not restore the wild type phenotype (Fig 5C). Conversely, complementation of the mutant strain with the four functional deletion mutants for the PGRS region of PE_PGRS33 restored the wild type phenotype, with the only exception of the *Mtb*Δ33::PE_PGRS33_401_ strain. Taken together these results suggest that the protein domain of PE_PGRS33 that contributes to *Mtb* entry in macrophages resides within the first 260 amino acids of the protein.

## Discussion

The appearance and expansion of PE_PGRS genes in certain mycobacteria has been linked to the acquisition of new pathogenetic properties that made these organisms capable of causing disease in mammals [4,9]. Despite the great interest raised by these findings, studies aimed at addressing the role of PE_PGRS proteins in *Mtb* biology and TB pathogenesis are still limited [34]. In a seminal work, the role of PE_PGRS33 was partially characterized in BCG, using a strain were the PE_PGRS33 was inactivated by random mutagenesis [19]. In this work, we extended these findings and further dissected the role of PE_PGRS33 in the virulent strain *Mtb*. The results obtained demonstrate that PE_PGRS33 is required for efficient *Mtb* cell entry in macrophages, but not in type II pneumocytes, while is dispensable for bacilli intracellular replication. PE_PGRS33-mediated entrance into macrophages is dependent upon the TLR2 receptor and this interaction occurs through the PGRS domain of the protein, specifically involving the first 260 amino acids of PE_PGRS33. These results indicate that PE_PGRS33 plays a crucial role during *Mtb* infection and provide experimental evidences that shed some light on the functional role of this protein in TB pathogenesis.

Lack of PE_PGRS33 was associated with an impaired ability of the *Mtb*Δ33 mutant strain to enter macrophages, probably because of a defect in bacterial attachment to host cells. This defect was not observed during infection of A549 pneumocytes, where *Mtb* entry is mediated by molecules different from those involved during macrophages entry [35], thereby suggesting a specific interaction of PE_PGRS33 with macrophage surface components. The reduction in intracellular CFUs measured for the *Mtb*Δ33 mutant was robust and similar to the reduction observed when the most important macrophage receptors involved in *Mtb* phagocytosis were blocked by specific antibodies [36]. Since *Mtb* enters macrophages by receptor-mediated phagocytosis [37] these results raised the possibility that PE_PGRS33 may directly interact with a macrophage receptor.

Several major host-receptors are involved in phagocytosis of *Mtb*, such as complement receptors (CR1, CR3 and CR4), the mannose receptor (MR) and the Fcγ receptors [37]. Fcγ is known to play a role in the presence of specific antibodies and entry via this receptor is expected to generate a vigorous host response [36]. Lipoarabinomannans, mannans and mannoproteins present on the *Mtb* surface are accessible to the MR and contribute to the adhesion of the bacilli to macrophages [38]. The MR also mediates uptake of mycobacteria other than *Mtb* [39], which do not express any PE_PGRS protein and therefore, in our view, it is unlikely that PE_PGRS33-mediated phagocytosis occurs through interaction with MR.

In previous studies [15] we showed that overexpression of PE_PGRS33 impacts on cell morphology and modifies the surface of bacilli. It may be hypothesized that lack of PE_PGRS33 may alter the proper conformation and/or the composition of mycobacterial surface constituents, such as for instance LAM or other non-proteinaceous components involved in *Mtb* cell entry and activation of pro-inflammatory cytokines secretion through CD14 and TLR2 [40–43]. In this scenario, the effects observed following overexpression of PE_PGRS33, or in its absence, might be considered simple epiphenomena. However, we did not observe any major difference in the cell morphology of the *Mtb*Δ33 compared with the wild type and complemented strains. It remains to be determined the causes of the differences observed between the mutants of PE_PGRS33 obtained in BCG [19] and *Mtb*, although immunoblot analysis with an anti-PGRS antibody of the total cell fractions of BCG and *Mtb* showed differences in the protein profile, suggesting that PE_PGRS expression and eventually localization in these two mycobacteria might be different [27].

The results obtained with the *Mtb*Δ33 complemented with the wild type gene PE_PGRS33 (*Mtb*Δ33::PE_PGRS33^HA^) and the *Mtb*Δ33::PE_1818c_, indicate that the PE domain of PE_PGRS33 does not contain the information sufficient to mediate PE_PGRS33-mediated entry in macrophages, in line with previous findings [19,23,33]. In an attempt to identify the domain, within the PGRS region, responsible for the observed phenotype a panel of functional deletion mutants expressing PE_PGRS33 protein missing fragments of increasing size of the C-terminal domain were generated (Fig 5). Interestingly, complementation of the wild type phenotype was obtained even with the mutant expressing the first 260 amino acids of the protein, which contains only a relatively small part of the PGRS domain. These results suggest that the PGRS domain residing between position 140 and 260 of the protein contains the information sufficient to interact with TLR2. The finding that the mutant *Mtb*Δ33::PE_PGRS33_401_ could only partially complement the phenotype, suggests that folding of the downstream domain of the PGRS is critical for the proper exposure of the first 260 amino acids of PE_PGRS33. Sequencing of the PE_PGRS33 gene in *Mtb* clinical isolates indicated that most of the genetic polymorphisms resides in the PGRS region, but mainly downstream of the first 260 amino acids [44–46], somehow supporting the key role of this domain. Dissecting the impact on function of these naturally occurring PE_PGRS alleles will be important to understand the role of the PGRS domain, from a pathogenetic and evolutionary perspective.

Using purified recombinant PE_PGRS33 protein obtained in *E*. *coli*, Basu *et al*. showed that PE_PGRS33 interacts directly with TLR2 ectopically expressed in RAW 264.7 or HEK293 cells; that triggering of TNF-α was dependent on the PGRS domain and that variations in the polymorphic repeats of the PGRS domain differentially modulated TNF-α secretion [23]. Recent data obtained in our laboratory, in the model of ectopic expression in *M*. *smegmatis*, demonstrated that PE_PGRS33-dependent TNF-α secretion in macrophages is dependent on TLR2 [33], thereby suggesting that PE_PGRS33 may indeed interact with TLR2 on the macrophage surface. Remarkably, the defect in macrophage entry observed for the *Mtb*Δ33 could not be observed when *pMM0* from TLR2-deficient mice were used, suggesting that PE_PGRS33-mediated entry of *Mtb* into macrophages is likely dependent on a direct interaction of PE_PGRS33 with TLR2. Unfortunately, the lack of any information on the structure of PE_PGRS33, or of any other PE_PGRS protein, prevents to hypothesize the mechanism of interaction with TLR2 [47].

The involvement of TLR2 in bacterial cell entry in macrophages has been proposed for several pathogens. Recognition through the CD14 of the *Porphyromonas gingivalis* fimbriae activates a TLR2-dependent signalling pathway promoting binding to and internalization by macrophages [48,49]. Interaction of these fimbriae with the CD14/TLR2 complex was shown to induce a PI3K-mediated inside-out signalling that activates the ligand-binding capacity of the CR3 and entry through CR3 was known to promote bacterial survival and virulence [31,48,50,51]. Similarly, it has been shown that *Bacillus anthracis* spores contain an hair-like nap formed by a glycoprotein that, by interacting with the CD14/TLR2 present on macrophages surface, activates the PI3K inside-out signalling pathway that enhances spore internalization by increasing the avidity of the Mac-1 integrin [52]. The relevance of this inside-out signalling, involving CD14, TLR2, PI3K and cytohesin and leading to the conversion of the low avidity CR3 into an active receptor, has been demonstrated also for mycobacteria [53,54]. The results of this study, indicating that treatment of *pMM0* with a PI3K inhibitor wortmannin or with the anti-CR3 antibody abolishes the PE_PGRS33-mediated entrance in macrophages (Fig 5), suggest that interaction of PE_PGRS33 with TLR2 activates the pro-adhesive pathway. While the mechanism of PE_PGRS33-mediated entrance needs to be better defined, it can be hypothesized that interaction of PE_PGRS33 with the TLR2 on macrophages in the early steps of infection activates an inside-out signalling which contributes to *Mtb* entry through the CR3 receptor.

This hypothesis is consistent with the TLR2-dependent pro-inflammatory properties of PE_PGRS33 that have been demonstrated by several studies [20,22,23,33]. In fact, it has been shown that *P*. *gingivalis* fimbriae induced two distinct TLR2 pathways mediating pro-inflammatory and pro-adhesive effects [30] and, more recently, that a fine tuning of the TLR2-CR3 crosstalk mediates inflammation and phagocytosis during *Francisella tularensis* infection [55]. In this scenario, PE_PGRS33 may be considered one of the *Mtb* proteins that plays a relevant role in the host-pathogen interplay, by fine tuning fundamental biological processes that can impact the outcomes of infection.

The relevance of PE_PGRS33 for *Mtb* pathogenesis can be inferred from the results obtained following genomic analysis of smooth tubercle bacilli [56]. The gene encoding PE_PGRS33 was found only in the MTBC genome but not in the smooth tubercle bacilli genome, and PE_PGRS33 was not found in *M*. *marinum*, despite the large number of PE_PGRS genes present in the genome of this mycobacterium [56]. The authors concluded that the PE_PGRS33 structural gene was inserted in MTBC next to the Rv1817 gene [56] and may thus have contributed to the acquistion of the specific pathogenic properties that made MTBC a highly successfull global pathogen.

In a previous study [11], we showed that the PGRS domain of PE_PGRS30 is involved in the arrest of phagosome acidification and in *Mtb* growth inside macrophages, while it is not involved in the entry of the bacteria inside macrophages. In this work, we demonstrate that the PGRS domain of PE_PGRS33 is involved in the bacterial entry in macrophages, but not in their intracellular replication. The reason why two very similar domains can be involved in so different cellular functions is still totally unknown and suggests that, despite the high similarity and redundancy of the GGA-GGN repeats, PE_PGRS proteins can be involved in a wide range of functions in *Mtb* pathophysiology.

## Supporting Information

S1 Fig Recombineering strategy to generate the knock out strain for the PE_PGRS33. (PDF)

S2 Fig schematic of the Rv1818c gene and PE_PGRS33 protein domains and demonstration of PE_PGRS33 expression in the complemented strain *Mtb* Δ33::PE_PGRS33^HA^. (PDF)

S1 Table Primers and plasmids used in this study. (DOCX)
