# Supplementary material for: PE_PGRS33 Contributes to Mycobacterium tuberculosis Entry in Macrophages through Interaction with TLR2
Source: PLoS One. 2016 Mar 15;11(3):e0150800. doi: 10.1371/journal.pone.0150800 (PMC4792380; doi:10.1371/journal.pone.0150800)
Supplement: S1 Fig — (PDF) [file pone.0150800.s001.pdf]

S1 Fig. Recombineering strategy to generate the knock out strain for the PE_PGRS33

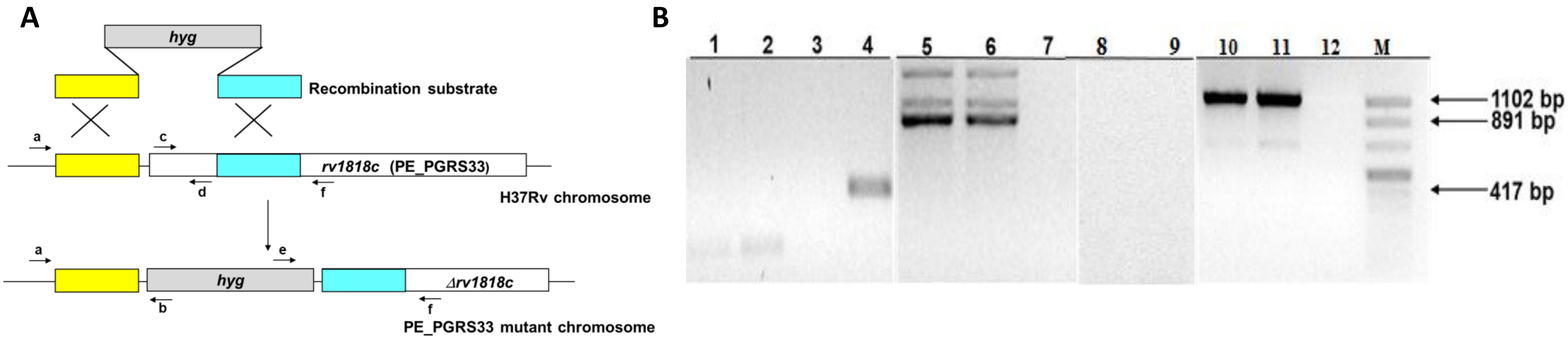

S1 Fig. Recombineering strategy to generate the knock out strain for the PE_PGRS33.

A) Schematic representation of the recombination event to obtain the deletion of the 5' region of *rv1818c*. The recombination targets are shown in yellow (*rv1818c* upstream region) and blue (*rv1818c* internal region), respectively. Integration results in the deletion of the first 561 bp of the gene including its translational start site. Primers used for the control PCR are shown. The figure is not drown to scale.

B) PCR performed on H37Rv recombinants obtained by recombineering. All reactions were performed on DNA extracted by two different recombinant mutants or wild type H37Rv.

Lines 1-4: reactions performed with oligonucleotides c and d; (1-2) recombinants clones, (3) no DNA, (4) H37Rv. Lines 5-8: reactions performed with oligonucleotides a and b; (5-6) recombinant clones, (7) no DNA, (8) H37Rv. Lines 9-12: reactions performed with oligonucleotides e and f; (9) H37Rv, (10-11) recombinant clones, (12) no DNA. Line M represents the DNA Molecular Weight Marker VIII from Roche Applied Science Arrows indicate the theoretical weight of the predicted amplified bands.

S1 Fig. Recombineering strategy to generate the knock out strain for the PE_PGRS33

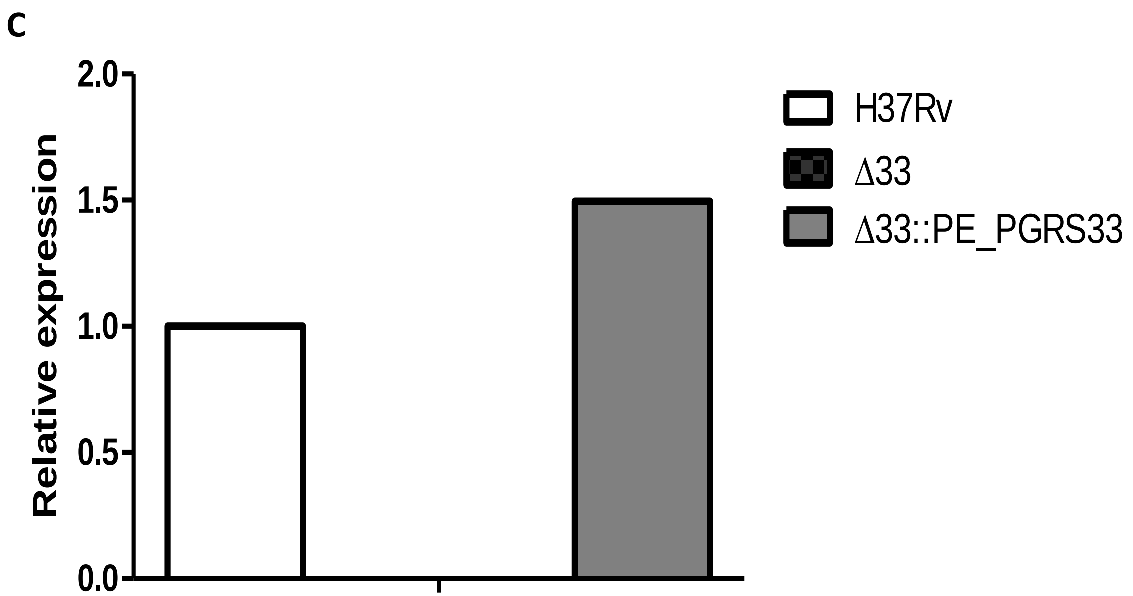

S1 Fig. Recombineering strategy to generate the knock out strain for the PE_PGRS33.

C) **Real-time quantitative PCR**: Total RNA was obtained from bacterial cell cultured in 7H9/ADC using RNAeasy plus mini kit (Qiagen, Valencia, CA) according to the manufacturer's directions. cDNA was prepared using iScript cDNA Synthesis Kit (Bio-Rad, Hercules, CA). Real-time quantitative PCR was done using the iCycler iQ System (Bio-Rad) and the iQ SYBR Green Supermix (Bio-Rad) in a final volume of 25 µL, starting with a 3-min template denaturation step at 95°C followed by 40 cycles of 15 s at 95°C and 1 min at 60°C. The primers used in this study to amplify the sequence of interest are: Rv1818cF:CTAGAGGGTGTGCTGACGT RV1818cR:CGCCGTTGCCGATCAAGATT. The experiments were repeated two or more times and each time the samples were run in triplicates. Relative quantification is based on the expression levels of a target gene *pe_pgrs33* versus the reference gene *ftsZ*. The results were analyzed as described previously using the Excel spreadsheet RelQuant (Bio-Rad) (Mozzetti et al., 2008).

Mozzetti S, Iantomasi R, De Maria I, Prislei S, Mariani M, Camperchioli A, Bartollino S, Gallo D, Scambia G, Ferlini C. (2008) Molecular mechanisms of patupilone resistance. Cancer Res. 2008 Dec 15;68(24):10197-204. doi: 10.1158/0008-5472.CAN-08-2091.
