# Supplementary material for: PE_PGRS33 Contributes to Mycobacterium tuberculosis Entry in Macrophages through Interaction with TLR2
Source: PLoS One. 2016 Mar 15;11(3):e0150800. doi: 10.1371/journal.pone.0150800 (PMC4792380; doi:10.1371/journal.pone.0150800)
Supplement: S2 Fig — (PDF) [file pone.0150800.s002.pdf]

S2 Fig. Schematic of the Rv1818c gene and PE_PGRS33 protein domains and demonstration of PE_PGRS33 expression in the complemented strain *Mtb* Δ33::PE_PGRS33^{HA}

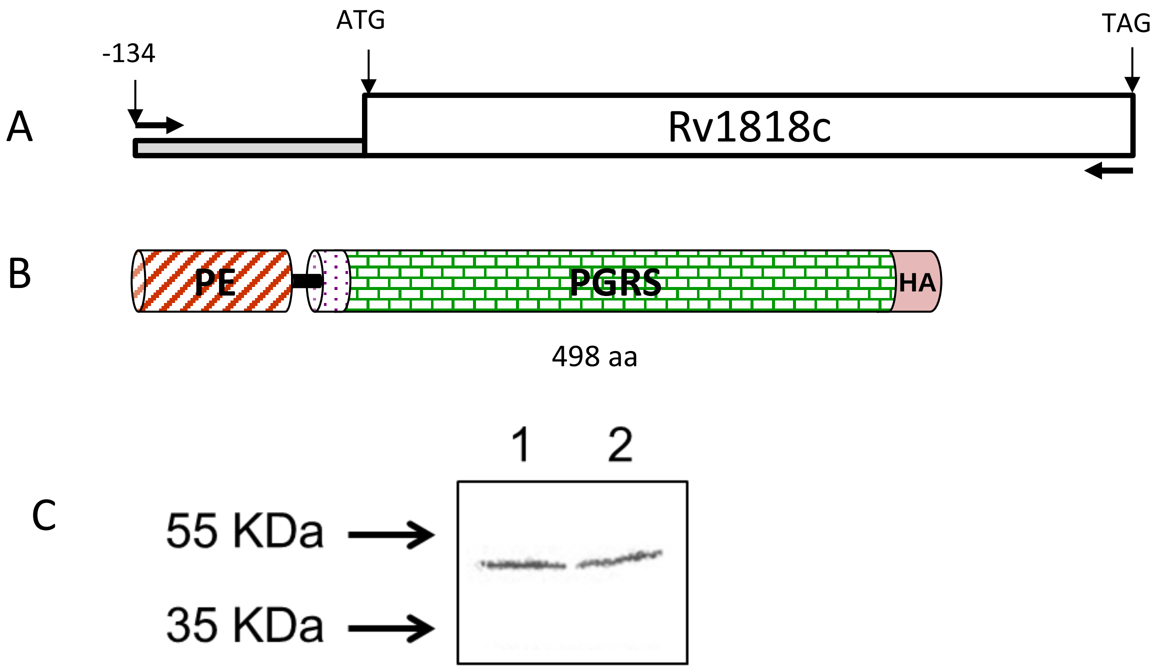

S2 Fig. Schematic of the Rv1818c gene and PE_PGRS33 protein domains and demonstration of PE_PGRS33 expression in the complemented strain *Mtb* Δ33::PE_PGRS33^{HA} .

A) Schematic representation of the *Mtb* chromosomal region introduced in pMV306 containing the entire *rv1818c* fused with the sequence encoding for the HA epitope and preceded by its own promoter; B) schematic showing the major domains of the PE_PGRS33^{HA} used for complementation studies; C) lane 1: immunoblot confirming the expression of PE_PGRS33^{HA} in *Mtb*Δ33 after its complementation (*Mtb* Δ33::PE_PGRS33^{HA}); lane 2, a previously characterized merodiploid *Mtb* strain expressing PE_PGRS33^{HA} was used as a positive control (15).

Bacterial cells were mechanically disrupted using a Mini-Beadbeater (BioSpec Products). SDS-PAGE was performed according to standard protocols. Briefly, proteins were separated on 10%, polyacrylamide gels, and subsequently transferred to polyvinylidene fluoride membranes (PVDF; Bio-Rad). Proteins were visualized by immunoblotting using monoclonal antibodies directed against the HA epitope (Anti-HA.11; Covance, dilution 1:2000) as primary antibody and goat anti-mouse (Santa Cruz Biotechnology; dilution 1:2000) horseradish peroxidase conjugates as secondary antibody. Chemoluminescent signal was developed using the West Dura Signal Kit (Pierce). Image acquisition was performed using a Versadoc Imaging System (Bio-Rad).
