# Supplementary material for: PE_PGRS33 Contributes to Mycobacterium tuberculosis Entry in Macrophages through Interaction with TLR2
Source: PLoS One. 2016 Mar 15;11(3):e0150800. doi: 10.1371/journal.pone.0150800 (PMC4792380; doi:10.1371/journal.pone.0150800)
Supplement: S1 Table — (DOCX) [file pone.0150800.s003.docx]

S1Table. **Primers and plasmids used in this study**

| PE_PGRS33 gene fragment | Forward primer | Reverse primer | Plasmid | Name of the strain |
| --- | --- | --- | --- | --- |
| PE_PGRS33 | **1818PR5Hn**  5’ACAAGCTTGCCGGGACGAGCTTCCCAAATCGG 3’  HindIII | **18c3AXb**  5’ ACTCTAGACGGTAACCCGTTCATCCCGTTCTT 3’  XbaI | Pmv10808 | **Mtb Δ33::PE_PGRS33** |
| PE_PGRS33 _472_ | **PG335Hn-338bp**  5’ACAAGCTTTTCCTCGACGAAAGTACCTCGGCG 3’  HindIII | **PG333Xb472**  5’ ACTCTAGACCCGGCGTTGCCGCCGTTGCCGCC 3’  XbaI | Pmv120808 | **Mtb Δ33::PE_PGRS33_472_** |
| PE_PGRS33 _401_ | **PG335Hn-338bp**  5’ACAAGCTTTTCCTCGACGAAAGTACCTCGGCG 3’  HindIII | **PG333Xb401**  5’ ACTCTAGAGCCGCCGTTTCCGCCGGCGCCGCC 3’  XbaI | Pmv12813 | **Mtb Δ33::PE_PGRS33_401_** |
| PE_PGRS33 _341_ | **PG335Hn-338bp**  5’ACAAGCTTTTCCTCGACGAAAGTACCTCGGCG 3’  HindIII | **PG333Xb341**  5’ ACTCTAGACCCGCCGGACCCCCCGGCACCGCC 3’  XbaI | Pmv12812 | **Mtb Δ33::PE_PGRS33_341_** |
| PE_PGRS33 _260_ | **PG335Hn-338bp**  5’ACAAGCTTTTCCTCGACGAAAGTACCTCGGCG 3’  HindIII | **PG333Xb260**  5’ ACTCTAGAGCCGGCACCGCCCACGCCGAAGAA3’  XbaI | Pmv10738 | **Mtb Δ33::PE_PGRS33_260_** |
| PE__rv1818c_ | **PErv1818c5Xb**  5’GCTCTAGAATGTCATTTGTGGTCACGATCC 3’  XbaI | **PERv1818c3**  5’ AAGGTACCTACCCATGGTTAATTAAGGATCCATTGCCGATCAAGTTCCG 3’  BamHI NcoI PacI | Cascioferro *et al,* 2011 | **Mtb Δ33::PE­__rv1818c_** |
